# Effect of Self-Healing by Dicyclopentadiene Microcapsules on Tensile and Fatigue Properties of Epoxy Composites

**DOI:** 10.3390/ma16145191

**Published:** 2023-07-24

**Authors:** Abhishek Pandey, Atul Kumar Sharma, Dharmendra Kumar Shukla, Kailash Narayan Pandey

**Affiliations:** 1 Mechanical Engineering Department, Motilal Nehru National Institute of Technology Allahabad, Prayagraj 211004, India; atulsharma19090@gmail.com (A.K.S.);; 2 Mechanical Engineering Department, ABES Engineering College, Ghaziabad 201009, India; 3 Mechanical Engineering Department, Indian Institute of Technology Jodhpur, Jodhpur 342037, India

**Keywords:** self-healing composites, polymer composites, mechanical characterization, tensile properties, fatigue life

## Abstract

Microcapsules of urea-formaldehyde (UF) containing dicyclopentadiene (DCPD) were synthesized by the in situ polymerization technique for self-healing of epoxy. The dispersion of microcapsules in the epoxy matrix was achieved using ultrasonication. Composites of epoxy, having 0.5, 1.0, 1.5, and 2.0 wt.% of microcapsules capable of self-healing, were prepared. The shape and size of the microcapsules were determined by field emission electron microscopy. Spherical capsules of DCPD, with an average diameter of 172 nm, were obtained. Investigation of tensile properties indicated a decrease in the tensile modulus with an increase in wt.% of microcapsules. There was a reduction of 22%, 27%, 39%, and 30% in the elastic modulus of composites for 0.5, 1.0, 1.5, and 2.0 wt.% of microcapsules, respectively. Tensile strength was found to increase with an increase in wt.% of microcapsules. The tensile strength of the composites increased by 33%, 20%, 8%, and 21% for 0.5, 1.0, 1.5, and 2.0 wt.% of microcapsules, respectively, in comparison with that of neat epoxy. The fatigue life of composites was investigated by conducting uniaxial tension–tension fatigue tests at constant stress amplitudes of 20, 25, 30, and 35 MPa, at a constant stress ratio (R = 0.1) and a frequency of 3 Hz. The fatigue life of composites increased with an increase in wt.% of microcapsules in comparison with that of neat epoxy. It was found that the fatigue life of the composites decreased with 1.5 and 2.0 wt.% of microcapsules in comparison with composites with 0.5 and 1.0 wt.% of microcapsules. The fracture surfaces of the tested samples were examined with the help of scanning electron microscopy (SEM) to understand the various mechanisms responsible for the change in modulus, strength, failure strain, and fatigue life of composites.

## 1. Introduction

The notable characteristics, such as being lightweight, high strength-to-weight ratio, stiffness properties, low cost, and high resistance to environmental degradation, make polymers and polymer composites efficient materials to be used in a variety of applications [1]. Nevertheless, these materials are susceptible to damage induced by mechanical, chemical, and thermal factors, or a combination of these factors. This damage could result from the formation of microcracks deep within the structure, where detection and external intervention are difficult or impossible, and conventional repair methods are not useful for healing invisible microcracks within the structure during its service life.

Various methods have been suggested for incorporating techniques to heal the damage in materials while undergoing service. White et al. [2] investigated ring-opening metathesis polymerization (ROMP) of dicyclopentadiene for self-healing of structural polymeric materials. Healing was triggered by the rupture of microcapsules and the release of microencapsulated DCPD into the crack. Self-healing polymeric composites with microencapsulated healing agents offer great potential for providing long-lived structural materials [3]. Yin et al. [4] reported the healing of an epoxy matrix by dispersing microcapsules filled with epoxy as a healing agent in it. The effect of the size of microcapsules on the healing of epoxy was investigated by Rule et al. [5]. The amount of liquid delivered to a crack face increases linearly with the increase in the diameter of microcapsules at a given wt.% of microcapsules. The essential and necessary conditions for the incorporation of healing agents include low viscosity and rapid consolidation of the healing material [6]. The concept of self-healing polymeric materials was proposed as a means of healing invisible microcracks to extend the working life and safety of the polymeric components [7].

Several encapsulation techniques for manifesting self-healing capabilities of epoxy are reported in the literature [8,9]. The effect of the mixing method and emulsifying agents on encapsulation was studied for better performance of self-healing materials, and it was concluded that the type of emulsifier and ultrasonication technique used determines the yield of microcapsules. The mixing rate is responsible for the amount of healing material, and the diameter of the capsules formed [10]. Khan et al. [11] used a relatively newer technique for self-healing using dual-component microcapsules. Microcapsules with polymethyl methacrylate (PMMA) as shell-wall material and epoxy resin and hardener as core materials were synthesized. The performance of metal–epoxy composite lap joints was investigated. The composition of microcapsules is one of the deciding factors in the self-healing capabilities of epoxy composites. The shell ruptures in the event of failure, and the healing material present in the shell flows in to heal the epoxy composites. Ahangaran et al. [12] used a dual-component system of microcapsules with a PMMA shell and epoxy prepolymer 3-aminomethyl-3,5,5-trimethylcyclohexylamine and pentaerythritol tetrakis (3-mercapto propionate) as a healing agent. They used ionic and polymeric emulsifiers separately to prepare microcapsules. Recently, Sun et al. [13] reported the use of organic phenol formaldehyde as shell material and DCPD as healing material for the healing of epoxy (E51). Self-healing systems using microencapsulation techniques are relatively better and versatile. The inclusion of microcapsules in a polymer matrix using commonly used techniques does not change the existing structure of the matrix.

The techniques employed for the development of healing ability in materials started with the use of the hollow glass fiber system. The other two methods, microvascular networks (MVNs) and microencapsulated systems (MECs), were developed simultaneously with a focus on MVNs due to their capability of healing more than once at the same site. The key challenge of continuous delivery of the healant was addressed in the MVN-based system. The MEC-based system gained momentum due to blockage and flow-related issues of the healant in the network. The improvement in life at the cost of strength needed to be balanced, and HGFs and MVNs failed to address this issue. This led to focused research on MECs to increase the life of components.

Sharma et al. reported an increase in the fracture toughness of healed composites of UF-DCPD-epoxy [14]. Shinde et al. investigated the SLA (stereolithography) method to fabricate components, and reported relatively poor mechanical strength compared with conventional methods [15]. Kontiza et al. investigated the healing efficiency of UF-DCPD microcapsules embedded in CFRPs (carbon-fiber-reinforced polymers) by various methodologies, and reported a reduction in the crack volume for the DCB test of up to 45% and mechanical strength restoration above 50% [16]. Cioffi et al. also discussed different mechanisms and schemes for self-healing, along with the dynamic and mechanical behavior of materials [17].

Recently, Kumar et al. [18] investigated DCPD as a healing agent with or without microcapsules. The load-carrying capacity was found to be increased. The study focused mainly on incorporating a healing mechanism that enhances the life of polymer composites without compromising mechanical integrity for its usage in structural applications. Jahadi et al. [19] proposed a new model for the evaluation of damage in two-component MECs based on polymer composites, and reported an increase in tensile strength due to an increase in interfacial strength, fracture energy, and Young’s modulus.

To the best of our knowledge, the microencapsulated system of self-healing still lags in the characterization of fatigue strength. We have investigated the tensile strength and fatigue life of epoxy and epoxy–DCPD composites with a change in wt.% of UF-DCPD microcapsules. This paper also explores the mechanisms responsible for the changes in tensile properties and fatigue life of composites.

## 2. Experimental

### 2.1. Material System

#### 2.1.1. Microcapsules

The materials used for the in situ polymerization of DCPD microcapsules are ethylene maleic anhydride (EMA) copolymer, dicyclopentadiene (DCPD), urea, ammonium chloride, formaldehyde, sodium hydroxide, hydrochloric acid, 1-octanol, and resorcinol. The ethylene maleic anhydride (EMA) copolymer was obtained from Vertellus Specialties Inc., Ledgewood, NJ, USA. Dicyclopentadiene (DCPD) was procured from Alfa Aesar, Thermo Fisher Scientific India Private Limited, Mumbai, India. Urea, ammonium chloride, formaldehyde, sodium hydroxide, hydrochloric acid, and 1-octanol were obtained from Thomas Baker (Chemicals) Pvt. Ltd., Mumbai, India. Resorcinol was obtained from Spectrochem Pvt. Ltd., Mumbai, India. Bis(tricyclohexylphosphine)benzylidene ruthenium (IV) dichloride, a Grubbs’ catalyst [20], and paraffin wax were procured from Sigma-Aldrich, Bengaluru, India.

#### 2.1.2. Matrix

The matrix material used in the present study was epoxy (bisphenol-A), synthesized using Araldite LY556 and hardener HY951, with densities of 1.17 g/cc and 0.98 g/cc, respectively, at a temperature of 25 °C. It was obtained from Huntsman Advanced Materials, New Delhi, India. Acetone, a solvent for epoxy, with a purity of 99%, was obtained from Rankem Chemicals (RFCL Limited), Peddapalli, India.

### 2.2. Preparation of Microcapsules

**Figure 1:**
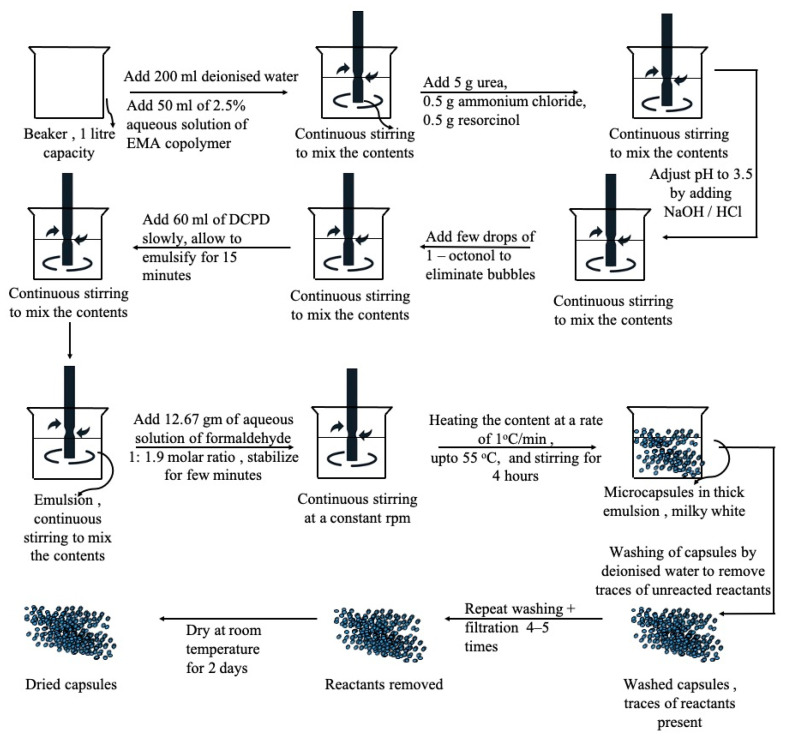
Process of synthesis of microencapsulation of DCPD [3].

Microcapsules were fabricated through in situ polymerization [3,21]. A flow diagram illustrating the synthesis of microcapsules is provided in Figure 1. To prepare the microcapsules, 50 mL of a 2.5 wt.% aqueous solution of EMA copolymer, 5.0 g of urea, 0.5 g of ammonium chloride, and 0.5 g of resorcinol were mechanically mixed with 200 mL of deionized water at room temperature. The resulting solution was placed in a water bath at room temperature and agitated using a digital shear mixer (Remi, RQT 134 H/D) at 2000 rpm. During agitation, the pH of the solution was adjusted to 3.5 by gradually adding sodium hydroxide (NaOH) and hydrochloric acid (HCl). A few drops of 1-octanol were added to prevent bubble formation on the surface.

A slow stream of 60 mL of DCPD was added to the solution to form an emulsion, and this was allowed to stabilize for the next 15 min to ensure complete emulsification. After stabilization, 12.67 g of an aqueous solution of formaldehyde was added to achieve a 1:1.9 molar ratio of formaldehyde to the solution. The emulsion was covered and heated on a hot plate, increasing the temperature at a rate of 1 °C per minute, until reaching 55 °C [3].

After continuous agitation for 4 h, the mixer and hot plate were switched off. The suspension was cooled to ambient temperature, and microcapsules of UF and DCPD were separated under vacuum using a coarse-fritted filter. The obtained microcapsules were rinsed with deionized water 3 to 4 times to remove any traces of unreacted constituents and then air-dried for 24–48 h. The Grubbs’ catalyst was re-crystallized by freeze-drying and cast into wax [22]. A 5 wt.% of the catalyst was mixed with the DCPD microcapsules.

#### Mechanism of Self-Healing

**Figure 2:**
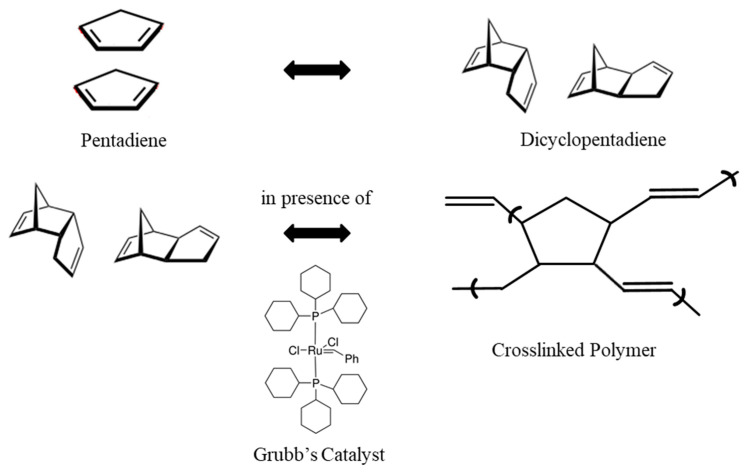
Mechanism of DCPD polymerization [23].

Poly-dicyclopentadiene (PDCPD) is a highly cross-linked polymer formed through ring-opening metathesis polymerization (ROMP) of dicyclopentadiene, widely used in high-performance composites. Metathesis reactions are chemical reactions in which two hydrocarbons (HCs) are converted into two new hydrocarbons by exchanging carbon–carbon bonds. The ROMP process involving metathesis is facilitated by the ruthenium-based Grubbs’ catalysts [23]. The sequence of reactions is depicted in Figure 2. In the case of DCPD, there are two available functional groups: the double bond of the norbornene ring and the double bond of the cyclopentene ring. It is well known that ROMP initially occurs at the highly strained norbornene ring, followed by the cyclopentadiene unit, resulting in a densely cross-linked polymer structure [24].

UF-DCPD microcapsules, along with ruthenium-based Grubbs’ catalysts, are embedded in the epoxy matrix. The catalyst triggers the polymerization of DCPD in the presence of a crack. Capillary action is responsible for the transport of DCPD to the crack site. The polymerization of DCPD leads to the bonding of the two crack faces. Microcapsule-based healing is an extrinsic healing system that requires a trigger to initiate the healing process. The mechanism is initiated by the rupture of the crack, which occurs due to damage or loading. The rupture of the microcapsules releases DCPD (healant). DCPD activates ring-opening metathesis polymerization. The energy required for the initiation of ring-opening and polymerization in DCPD is provided by the rupture of the microcapsules. The energy stored as ring strain (~27.2 kcal/mol) facilitates easy ring-opening and promotes subsequent polymerization, while substituents prevent the secondary metathesis of the polymer backbone [22].

### 2.3. Composite Preparation

**Figure 3:**
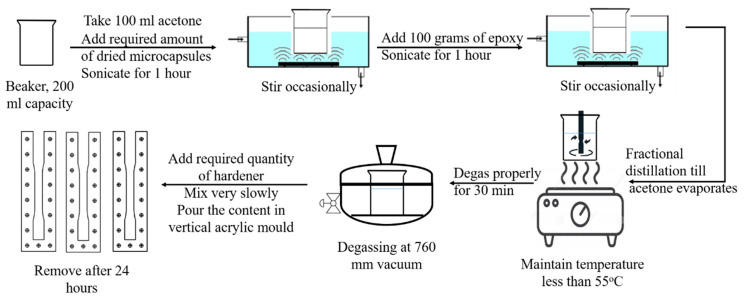
Fabrication process of composites.

Composites containing DCPD microcapsules in epoxy were fabricated using the in situ polymerization technique. Figure 3 illustrates a schematic of the composite fabrication process. The desired quantity of microcapsules was mixed with acetone, and the mixture was sonicated for an hour using a 230 W-rated bath sonicator operating at 50 Hz. The mixture was manually stirred at regular intervals to ensure proper dispersion. The required amount of epoxy resin (LY556) was added to the sonicated mixture, which was then sonicated for an additional hour. After sonication, the solvent (acetone) was removed through a partial distillation process.

Once the acetone was removed, the mixture was placed under vacuum for half an hour to eliminate any trapped gases. The hardener (HY951) was added to the mixture in a ratio of 1:10 by weight of epoxy resin and gently mixed. The resulting mixture was poured into a vertical acrylic mold and left to cure at room temperature for 24 h. The diamine hardener initiates crosslinking of epoxy chains, leading to the gelling and solidification of the liquid epoxy resin. This process is known as epoxy curing. After the curing process at room temperature, the composite sheets were removed from the mold. Composite sheets with 0.5, 1.0, 1.5, and 2.0 wt.% of microcapsules were fabricated.

### 2.4. Characterization

#### 2.4.1. Microcapsules

Images of microcapsules, captured using a field emission scanning electron microscope (FESEM, Sigma 300, Carl Zeiss, Oberkochen, Germany), were analyzed to investigate the shape and size of the microcapsules. To enable electrical conduction on the surface, the microcapsules were initially coated with gold. FESEM microscopy was performed with an accelerating voltage of 5 kV.

#### 2.4.2. Composites

The composites were examined under a scanning electron microscope (SEM, EVO 50, Carl Zeiss) to observe the dispersion of microcapsules in the epoxy. The samples, with dimensions of 13 mm × 5 mm × 5 mm, were prepared by machining and gold-coated to facilitate electrical conduction on the surface. The accelerating voltage was set at 10 kV.

### 2.5. Mechanical Characterization

#### 2.5.1. Tensile Properties

**Figure 4:**
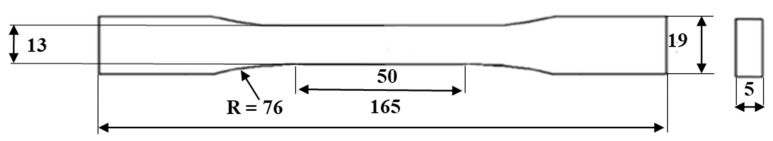
Tensile and fatigue test specimen (dimensions in mm) [25].

Tensile properties were determined by conducting a uniaxial tensile test following ASTM standard D638 [25] on a servo-hydraulic UTM (Nano Plug ‘n’ Play, BISS, Bangalore, India) equipped with a 25 kN load cell. The tensile test was performed on flat dog bone specimens with the following dimensions: gauge length of 50 mm, width of the narrow section of 13 mm, thickness of 5 mm, overall length of 165 mm, and width of the grip region of 19 mm (see Figure 4).

These samples were directly cast to the specified shape and size. Tensile tests were conducted at a crosshead speed of 10 mm/min. The tensile modulus of the samples was calculated from the slope of the initial linear portion observed in the stress–strain curve of both the neat epoxy and composite samples using regression analysis. The maximum stress at failure and failure strain were calculated from the stress–strain data. At least five valid tests were conducted to calculate the average value of the tensile properties, along with a 95% confidence level. Only tests in which failure occurred in the gauge section were considered valid.

#### 2.5.2. Fatigue Life

Fatigue tests of epoxy and epoxy/UF-DCPD composites with 0.5, 1.0, 1.5, and 2.0 wt.% of DCPD microcapsules were conducted following ASTM standard D7791 [26]. The fatigue tests were performed on a Nano Plug ‘n’ Play machine equipped with a 25 kN load cell. Uniaxial tension–tension fatigue tests were conducted to investigate the fatigue life of the epoxy/microcapsule composites. The tests were conducted at constant stress amplitudes of 20, 25, 30, and 35 MPa, with a constant stress ratio (R = 0.1) and a frequency of 3 Hz. A low frequency of 3 Hz was chosen to avoid thermal effects on the test specimens. Sinusoidal waveforms were used for all the fatigue tests.

At least five samples were tested at each stress amplitude, and the average number of cycles to failure (N_f_) was determined. The N_f_ values, along with a 95% confidence level, were obtained for each weight percentage of microcapsules, and an S-N curve was plotted.

## 3. Results and Discussion

### 3.1. Size of Microcapsules

**Figure 5:**
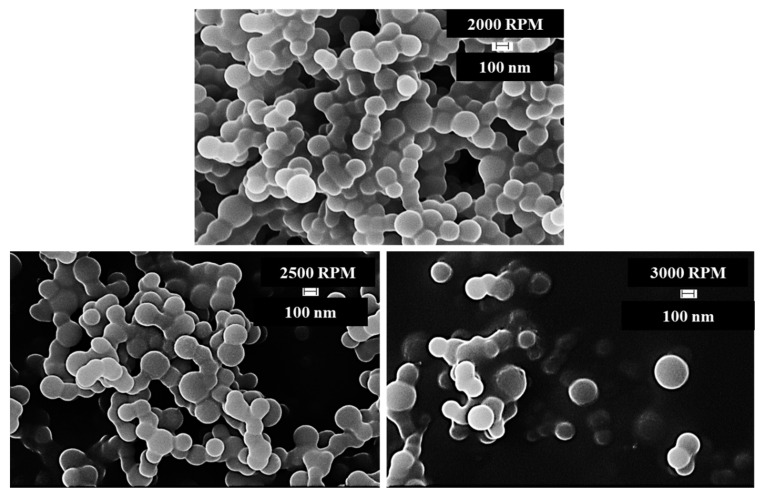
FESEM images of DCPD microcapsules synthesized at different agitation rates.

The shape and size of the DCPD microcapsules synthesized at different agitation rates were determined by analyzing FESEM images of the microcapsules. The microcapsules were fabricated using three different agitation rates: 2000, 2500, and 3000 rpm. FESEM images of the microcapsules prepared at these agitation rates are shown in Figure 5. It is evident from Figure 5 that the synthesized DCPD microcapsules are spherical in shape. The highest yield was observed at 2000 RPM, and the number of microcapsules and their diameter were found to be inversely proportional to the agitation rate [3]. The diameter of the microcapsules decreases as the agitation rate increases. However, the yield of microcapsules decreases at higher agitation rates.

More than a hundred microcapsules were analyzed using Image J software, an open-source software developed at the National Institute of Health and the Laboratory for Optical and Computational Instrumentation, University of Wisconsin, to determine the average size of microcapsules synthesized at different agitation rates. The average diameter of the microcapsules obtained at different agitation rates is presented in Table 1.

**Figure 6:**
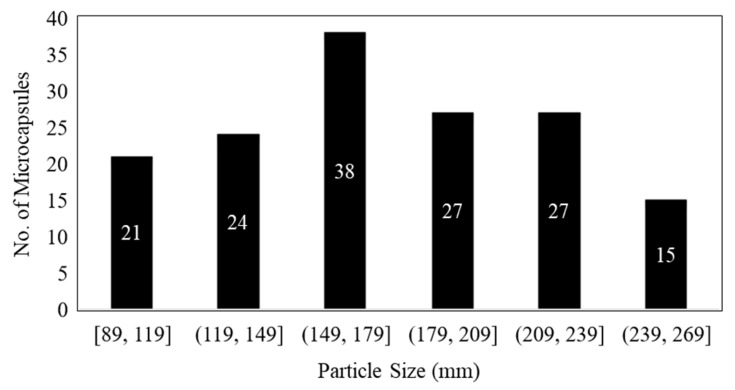
Average size of DCPD microcapsules at 2000 rpm.

Figure 6 illustrates the range of diameters of microcapsules and the number of microcapsules within each range for capsules synthesized at an agitation rate of 2000 rpm. Two batches of microcapsules were prepared at an agitation rate of 2000 rpm, labeled as 2000-1 and 2000-2 in Table 1. The average size of the microcapsules decreased by 20% and 25% at agitation rates of 2500 rpm and 3000 rpm, respectively, compared with the capsules obtained at 2000 rpm.

### 3.2. Dispersion of Microcapsules

**Figure 7:**
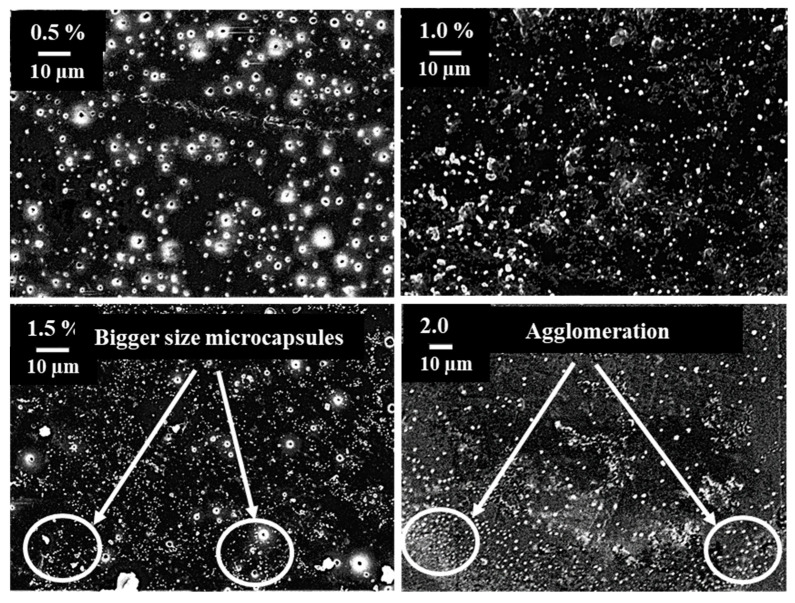
SEM image of composites showing dispersion for different wt. percentages.

SEM images of composites with 0.5%, 1.0%, 1.5%, and 2.0% wt.% of microcapsules are depicted in Figure 7. It is evident from Figure 6 that DCPD microcapsules are uniformly distributed throughout the matrix. A more uniform and proper dispersion is observed in the case of 0.5% wt.% microcapsules compared with the other weight percentages. As the wt.% of microcapsules increases, some agglomeration can be observed. In particular, larger microcapsules are observed in composites with 1.5% wt.% of microcapsules. The FESEM images indicate an increasing concentration of capsules from 0.5 wt.% to 2.0 wt.%, which may be the result of the proper distribution of microcapsules during the fabrication of composites at different weight percentages. However, some agglomeration of microcapsules is observed at higher weight percentages.

### 3.3. Tensile Properties

**Figure 8:**
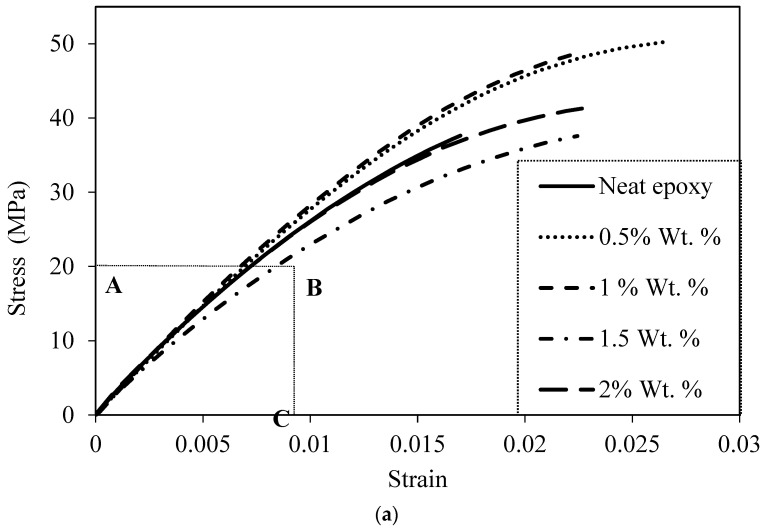

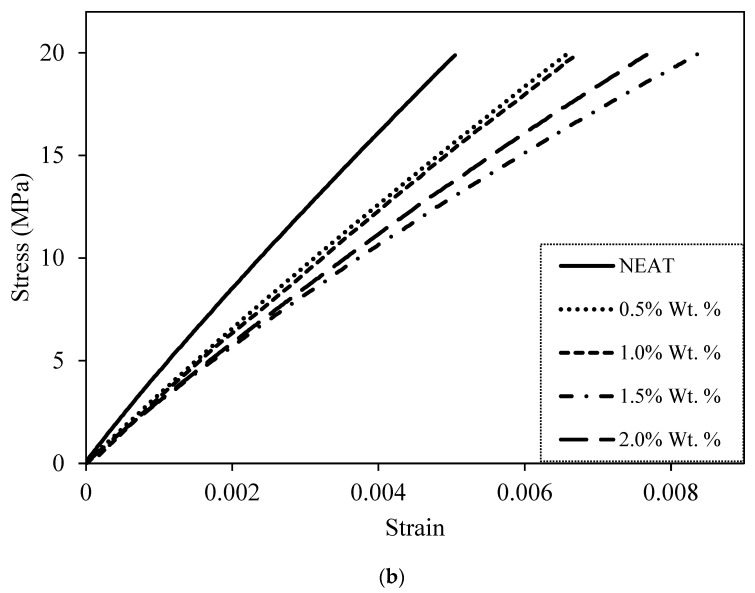
(**a**) Stress–strain diagrams of composites for different wt.% of microcapsules. (**b**) Initial linear portion of the stress–strain curve for the composites in region A,B,C of (**a**). (A—Stress up to 20 MPa, B—Strain up to 0.009).

The strain–stress curves of the neat epoxy and composites are presented in Figure 8a. The stress–strain curves of all the composites exhibited a linear behavior during the initial loading, from which the tensile modulus was determined using regression analysis. Figure 8b provides a modified view of the initial linear portion of Figure 8a, focusing on the best-fit linear portion of the stress–strain diagrams. It can be observed from Figure 8b that, as the weight percentage of the microcapsules increases, the slope of this initial linear portion decreases, indicating a decrease in the elastic modulus.

Figure 8a also illustrates that the neat epoxy has a significant nonlinear region. The nonlinear portion increases in the stress–strain curves of the composites. The composite with the 0.5% weight percentage of microcapsules exhibits the highest nonlinear portion. This nonlinear region begins at approximately 0.7% strain and extends up to the failure strain of 2.7%. The nonlinear portion decreases in composites with 1.0%, 1.5%, and 2.0% weight percentages of microcapsules compared with the composite with 0.5% microcapsules, but it remains considerably higher compared with that of the neat epoxy.

**Figure 9:**
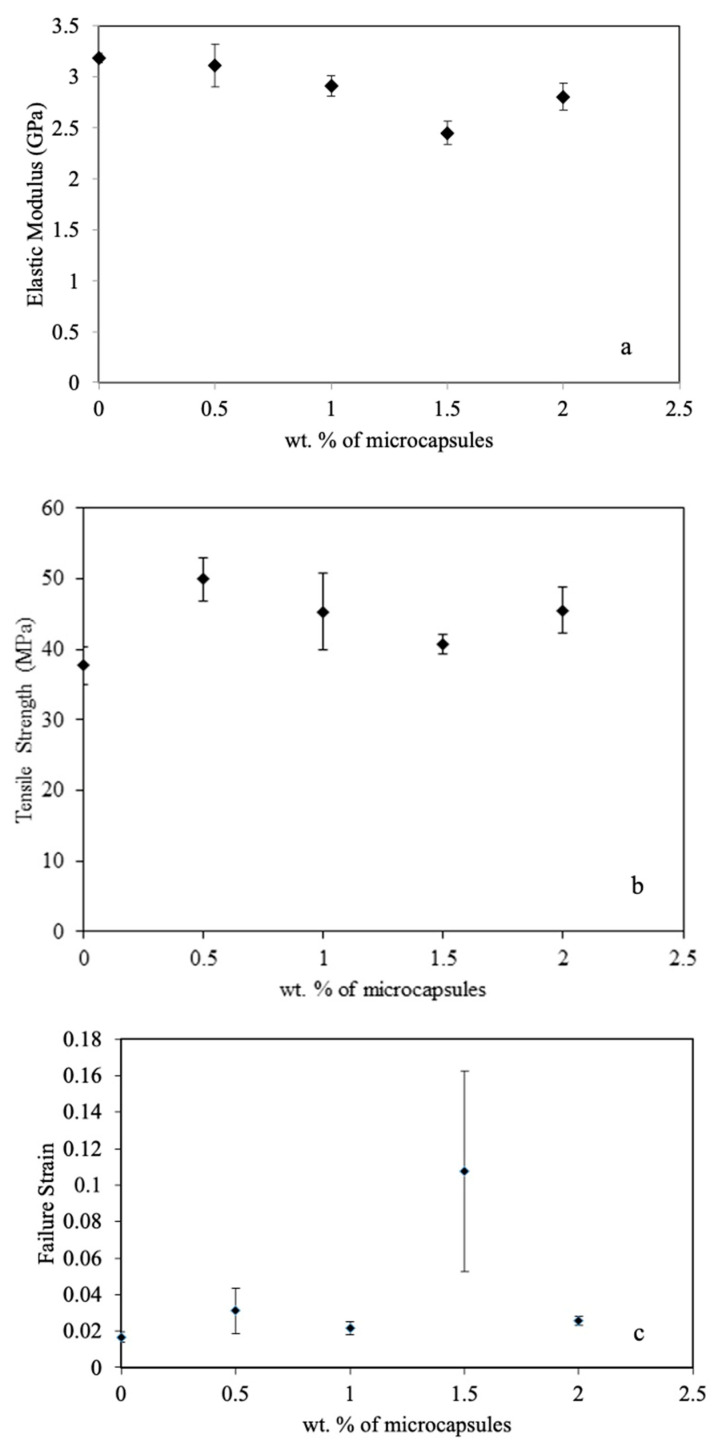
(**a**) Elastic modulus vs. wt.% of capsules, (**b**) tensile strength vs. wt.% of capsules, and (**c**) failure strain vs. wt.% of capsules.

Figure 9a illustrates the Young’s modulus of the composites as a function of the weight percentage of microcapsules. The Young’s modulus decreases by 22%, 27%, 39%, and 30% for 0.5%, 1.0%, 1.5%, and 2.0% weight fractions of microcapsules, respectively. This reduction in Young’s modulus can be attributed to the inclusion of soft microcapsules. Soft microcapsules do not provide reinforcement to the polymer chains against deformation, resulting in a decrease in the Young’s modulus of the composites. Similar behavior has been reported in the literature for the inclusion of soft rubber particles in epoxy [27,28,29].

Figure 9b displays the tensile strength of the composites as a function of the weight percentage of microcapsules. The tensile strength increases at the 0.5% weight percentage of microcapsules and decreases with further inclusion of microcapsules. However, the tensile strength of the composite remains higher than that of the neat epoxy at all weight percentages considered in the study. The tensile strength increases by 33%, 20%, 8%, and 21% for 0.5%, 1.0%, 1.5%, and 2.0% weight fractions of microcapsules, respectively. The tensile strength of the composites depends more on the load transfer between the matrix and reinforcement. The results indicate that microcapsules have a good ability to transfer load from the matrix.

Moreover, the release of self-healing material (DCPD) from the ruptured UF microcapsules delays the initiation and growth of cracks, thereby increasing both tensile strength and strain. The significant improvement in the tensile strength of composites at the 0.5% weight percentage of microcapsules can be attributed to the uniform dispersion of microcapsules. Additionally, the size of the microcapsules falls within the submicron range (172 nm), which eliminates the negative effect typically associated with rigid micron-sized particles on tensile strength [30]. The reduction in tensile strength of composites at higher weight percentages of microcapsules can be attributed to the agglomeration of microcapsules. Thus, the positive impact of microcapsules is counteracted by the negative effect of agglomeration. The substantial reduction in tensile strength of the composite at the 1.5% weight percentage of microcapsules is due to the presence of larger-sized microcapsules (as observed in SEM images in Figure 7).

The failure strain was determined by calculating the strain at the point of fracture in the tensile test. Figure 9c presents a comparison of the failure strains in the composites. The variation in failure strain of the composites with an increase in the weight percentage of microcapsules follows a similar trend to that of tensile strength. The only exception is the composite with the 1.5% weight percentage of microcapsules, which exhibits a significant increase (five times that of neat epoxy) in failure strain. On the other hand, an 87%, 30%, and 54% increment in failure strain was observed at 0.5%, 1.0%, and 2.0% weight fractions of microcapsules, respectively. The unprecedented increase in failure strain at the 1.5% weight percentage of microcapsules can be attributed to the release of a high amount of healing agent (DCPD) from the larger microcapsules. However, the healant does not have sufficient time to polymerize and heal the crack as the fracture occurs within a fraction of a minute.

### 3.4. Fatigue Life

**Figure 10:**
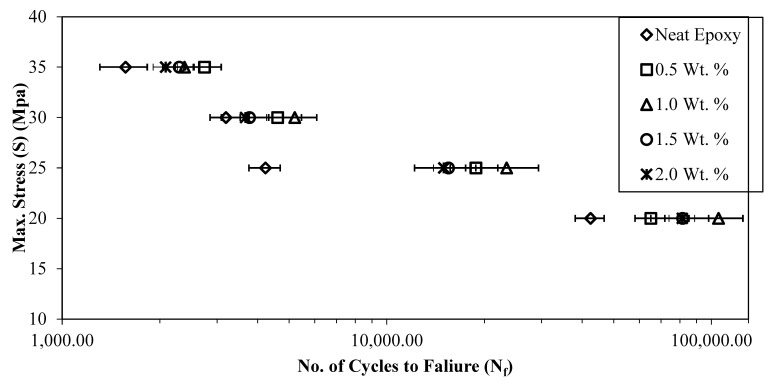
S-N curves of neat epoxy and epoxy–DCPD composites.

Uniaxial tension–tension fatigue tests were performed at constant stress amplitudes of 20, 25, 30, and 35 MPa and a frequency of 3 Hz to investigate the behavior of epoxy and epoxy–DCPD composites. The fatigue tests were conducted at a constant stress ratio (R) of 0.1. The stress levels were chosen to test the performance of the composites in both low-cycle and high-cycle fatigue. Figure 10 presents the S-N curves of epoxy and epoxy–DCPD composites with 0.5%, 1.0%, 1.5%, and 2.0% weight percentages of microcapsules. The figure is presented as a semi-log plot, with the maximum applied stress (S) on the y-axis and the average number of cycles to failure (N_f_) on the logarithmic x-axis. The horizontal error bars represent the 95% confidence level in the N_f_ data.

An improvement of 52%, 146%, 91%, and 90% in the average number of cycles to failure was recorded compared with neat epoxy at 0.5%, 1.0%, 1.5%, and 2.0% weight percentages of microcapsules, respectively, at a stress level of 20 MPa in the high-cycle fatigue region. In the transition region (at a stress level of 25 MPa), the maximum improvements in the average number of cycles to failure were observed (373%, 453%, 266%, and 254% at 0.5%, 1.0%, 1.5%, and 2.0% weight percentages of microcapsules, respectively), as shown in Table 2. At higher stress levels (30 and 35 MPa), the improvement in the average number of cycles to failure over that of neat epoxy ranged from 15% to 75%. The increase in fatigue life of the composites can be attributed to the healing and reinforcing effect of the microcapsules. Microcracks generated due to fatigue lead to the rupture of the capsules and the release of the healing agent. The healing agent has sufficient time to heal the microcracks at lower stress levels (20 MPa and 25 MPa), which is evident from the greater improvement in the fatigue life of the composites. On the other hand, at higher stress levels, the crack grows at a faster rate, giving less time for the healing agent to heal the crack, resulting in a lower improvement in the fatigue life of the composites.

It is evident from Figure 10 that N_f_ is maximum for composites with the 1.0% weight percentage of microcapsules at all stress levels except for 35 MPa. The decrease in N_f_ was less pronounced for composites with 1.5% and 2.0% weight percentages of microcapsules compared with composites with the 1.0% weight percentage of microcapsules. The decrease in fatigue life of the composites at 1.5% and 2.0% weight percentages of microcapsules could be attributed to the higher chances of capsule agglomeration at higher weight percentages. Microcapsules of larger size and agglomerated microcapsules act as stress raisers, resulting in higher actual stresses at the microscopic level than those calculated based on mechanics. Thus, an excessive amount of healing material does not contribute to a significant improvement in fatigue life.

**Figure 11:**
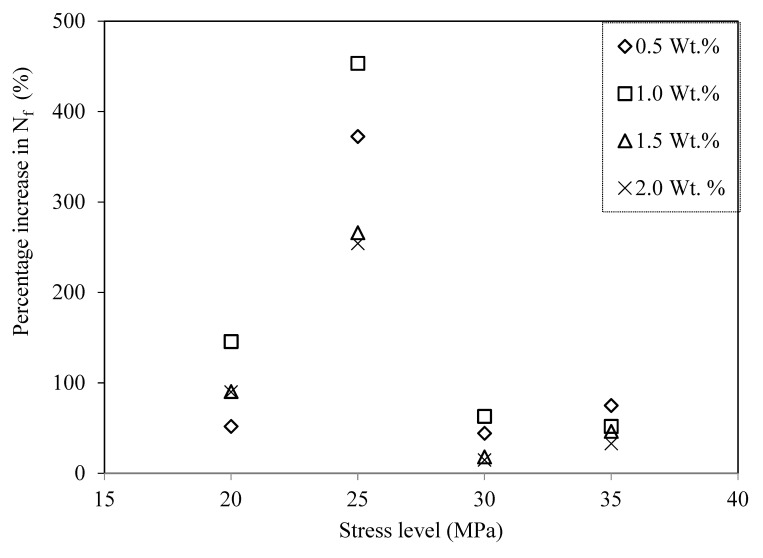
Percentage increase in number of cycles to failure (Nf) for epoxy/DCPD composites.

Table 2 provides the details of the fatigue test results conducted on self-healing composites. It lists the minimum, average, and maximum number of cycles to failure for the composites at a given stress level and weight percentage of microcapsules. Additionally, to highlight the improvement obtained in the average number of cycles to failure, it is plotted as a function of the stress level in Figure 11 for different weight percentages of microcapsules.

The decrease in the fatigue life of the composites at 1.5% and 2.0% weight percentages of microcapsules can be attributed to the higher chances of capsule agglomeration at higher weight percentages. Furthermore, an excessive amount of healing material does not contribute to the improvement of fatigue life.

At higher stress levels (30 and 35 MPa), the improvement in the average number of cycles to failure (N_f_) over that of neat epoxy ranged from 15% to 75%. Figure 11 clearly shows that N_f_ was maximum for composites with the 1.0% weight percentage of microcapsules. N_f_ decreased for composites with 1.5% and 2.0% weight percentages of microcapsules compared with composites with the 1.0% weight percentage of microcapsules.

### 3.5. Fractography of Fractured Surfaces

Resistance to crack propagation was found to be higher in composites with the 1.0% weight percentage of UF-DCPD microcapsules compared with composites with 0.5%, 1.5%, and 2.0% weight percentages. The study on particle dispersion suggested that the composite loaded with the 1.0% weight percentage exhibits a more uniform distribution of microcapsules compared with higher weight percentages. It is also essential to note that the fatigue life of the 1.0% loading is higher than that of the other composites. In this context, more focus is given to the fracture surface analysis of the 1.0% weight percentage of epoxy–DCPD composites. However, the analysis of fracture surfaces of the other composites (0.5%, 1.5%, and 2.0% weight percentages) under the loading condition of 35 MPa is also presented.

**Figure 12:**
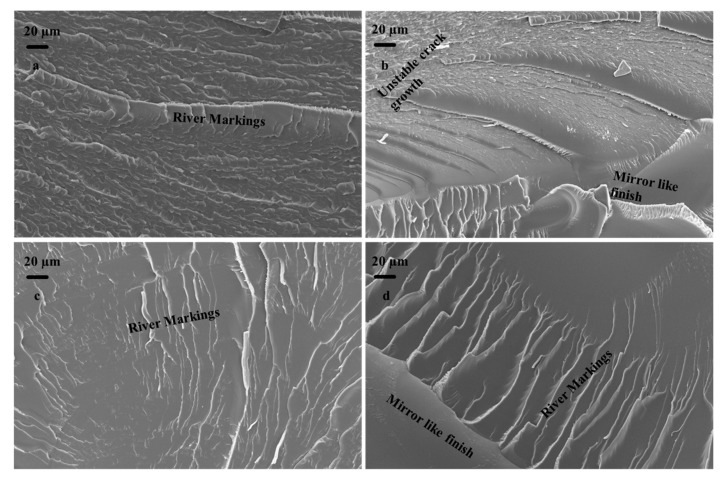
Fractured surfaces of neat specimen at (**a**) 20 MPa, (**b**) 25 MPa, (**c**) 30 MPa, and (**d**) 35 MPa.

SEM images of the fractured surfaces of the neat specimen at 20 MPa, 25 MPa, 30 MPa, and 35 MPa, respectively, are shown in Figure 12a–d. Various features such as river markings and a mirror-like finish are clearly visible in the images, confirming the failure phenomenon. The fractographic analysis of neat epoxy illustrates the development of characteristic features attributed to the epoxy. Primarily, the fracture surface of epoxy can be categorized into three different regions: (i) slow crack growth—mirror-like region, (ii) a smooth region associated with unstable crack propagation, and (iii) a three-dimensional rough surface related to unstable crack propagation. The fracture surface of neat epoxy demonstrates all three distinct features at different length scales [31,32].

**Figure 13:**
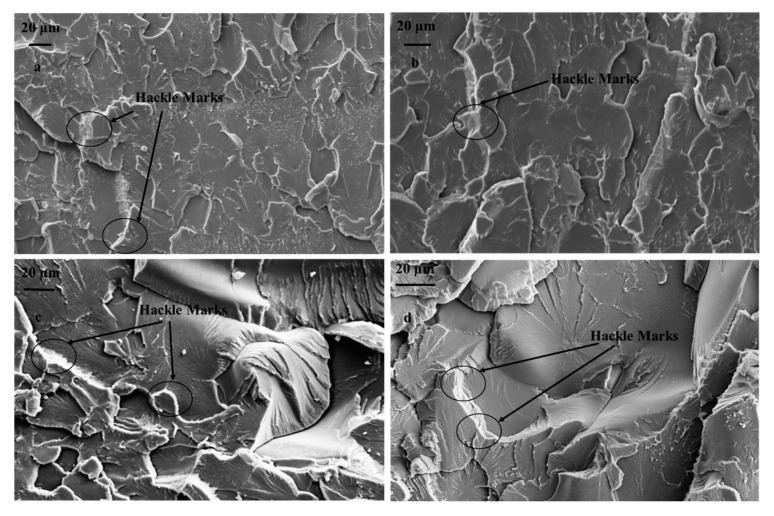
Fractured surfaces of composite having 1.0 wt.% capsules tested at (**a**) 35 MPa, (**b**) 30 MPa, (**c**) 25 MPa, and (**d**) 20 MPa.

SEM images of the fractured surfaces of composites filled with the 1% weight percentage of DCPD microcapsules are shown in Figure 13. The surfaces shown in Figure 13a–d were obtained as a result of fracture due to fatigue loading at maximum stresses of 35 MPa, 30 MPa, 25 MPa, and 20 MPa, respectively. It is evident from Figure 13a–d that changing the loading conditions significantly changed the fracture phenomenon. It can be observed from Figure 13 that the roughness of the fracture surface increases with the decrease in maximum stress. This indicates that the crack required higher energy to grow at lower loads. The microcracks were healed by the inflow of the healant DCPD due to the rupture of the microcapsules. At higher loads, effective healing could not be provided by the capsules as the crack extension took place at a faster rate, resulting in a smoother fracture surface.

**Figure 14:**
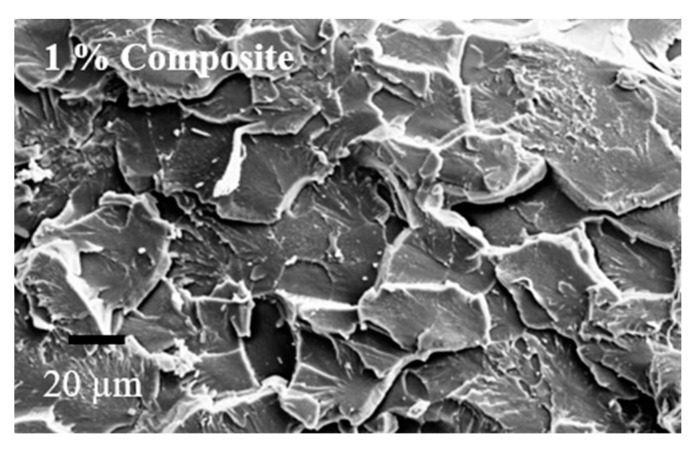
SEM image of fractured surface at 25 MPa.

The fracture mechanism of epoxy in the presence of fillers is primarily governed by the type of filler, their physicochemical properties (shape, size, functionality), volume fraction, etc. Such a fracture phenomenon mainly includes crack pinning, crack bowing, debonding, crack deflection, etc. [33,34]. In the context of microencapsulated self-healing reinforcement, crack pinning is the predominant fracture mechanism, but it is different from the classical crack pinning phenomenon in many aspects [21,35]. Similar failure mechanisms are reflected in our work. The fracture surfaces show the formation of hackle structures over the entire crack plane, as delineated in Figure 13a–d. Hackle structures are dominant compared with other mechanisms at lower fatigue loads, i.e., 20 MPa and 25 MPa (see Figure 14). For comparison, the fractured surfaces of the neat specimen are already shown in Figure 12.

The formation of hackle structures is facilitated due to the reconnection of two crack faces by step tail formation in the microencapsulant [21]. The formation of a tail in the case of the soft microencapsulant and hard spherical reinforcement can be clearly differentiated. However, such a tail formation does not take place in urea-filled self-healing microcapsules, and the formation of hackle marking is developed via the crack propagation along the equator of the particles. The hackle markings were found to be more prominent under the loading condition of 25 MPa compared with other loading conditions.

It is important to note here that an increase in the fatigue load can significantly change the speed of crack propagation. For instance, the fracture surface of 1.0 wt.% reinforced epoxy at 20 MPa of loading contains comparatively more mirror-like features, which correspond to the slow crack propagation phenomenon (Figure 13d). A further increase in loading has facilitated the formation of intense “river-like markings”, generated due to the crack propagation in two different planes. At 35 MPa, the fracture surface of the composite is dominated by crack bifurcation or unstable crack propagation (see Figure 13a). The formation of typical three-dimensional crack markings is attributed to the formation of secondary cracks in front of the primary crack, and the actual shape is defined by the relative velocities between the primary and secondary cracks.

**Figure 15:**
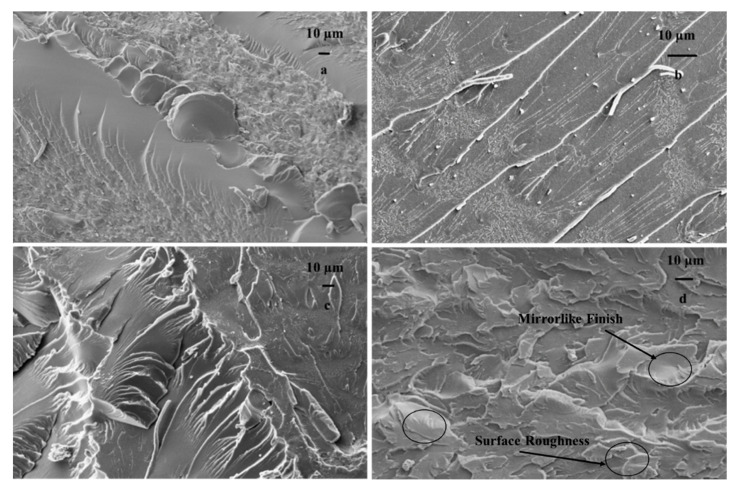
Fractured surface of samples tested at 35 MPa: (**a**) neat epoxy and composites, (**b**) 0.5 wt.%, (**c**) 1.0 wt.%, and (**d**) 1.5 wt.%.

In order to contemplate the fracture mechanism during the failure process, SEM analysis was conducted on the fractured surfaces of composites fractured at 35 MPa. Figure 15 exhibits the fracture surface of neat epoxy in addition to the composites reinforced with 0.5, 1.0, and 1.5 wt.% loadings. It is evident from Figure 15b–d that reinforcing DCPD in the epoxy matrix has facilitated the formation of a rougher surface, which is found to be augmenting with the increase in microcapsules. As elaborated earlier, the increase in wt.% of microcapsules causes the formation of agglomerates due to the cohesive force among the DCPD capsules. The effect of such agglomerates is clearly visible on the fracture surface of the corresponding composite system, which indicates that an increase in the wt.% of DCPD renders more brittle-like fracture characteristics, such as a mirror-like finish and an increase in surface roughness.

### 3.6. Correlation of Fatigue Strength Coefficient and Fatigue Strength Exponent by Basquin Equation

Basquin proposed a mathematical power law that describes the relationship between the applied stress (S) and the number of cycles to failure (Nf). The equation used to design the object using the S-N curve for a finite number of cycles to failure (N_f_ < 10^6^) is given by [36]:

S = ANf^b^

where S is the applied stress, N_f_ is the number of cycles to failure, A is the fatigue strength coefficient (FSC), and b is the fatigue strength exponent (FSE).

The FSC and FSE are obtained through regression analysis for all weight percentages considered in the present work. The values of FSC (A) and FSE (b) for neat epoxy and epoxy–DCPD composites with 0.5%, 1.0%, 1.5%, and 2.0% weight percentages of microcapsules are obtained by fitting the above equation with the data from Figure 11. The values of FSC and FSE for neat epoxy and composites are listed in Table 3.

An increment of 21% in FSC (A) over that of neat epoxy was recorded for composites with the 0.5% weight percentage of DCPD microcapsules. The value of FSC (A) decreases for composites with higher weight percentages of microcapsules. The decrease in FSC (A) can be attributed to the agglomeration of DCPD microcapsules at higher weight percentages of microcapsules. The agglomeration is clearly observed in Figure 6 for composites with higher weight percentages of microcapsules. The decrease in tensile properties at higher percentages can also be attributed to the decrease in FSC at higher weight percentages of UF-DCPD microcapsules in composites. The decrease in the FSC of UF-DCPD composites compared with that of neat epoxy might be due to the inclusion of soft microcapsules as filler particles. The soft microcapsules are unable to share the load and deter the propagating crack.

A reduction of 5% in FSE (b) compared with that of neat epoxy was observed for composites with the 0.5% weight percentage of DCPD microcapsules. The value of FSE (b) increased in comparison with that of neat epoxy with the further addition of microcapsules, i.e., for composites with higher weight percentages of microcapsules.

## 4. Conclusions

The self-healing behavior of composites containing DCPD in the epoxy matrix was investigated using DCPD microcapsules. The shape and size of the fabricated microcapsules were determined by taking FESEM images. It was observed that the microcapsules were spherical in shape, with an average diameter of 172 nm at an agitation rate of 2000 rpm. Furthermore, the average size of microcapsules decreased by 20% and 25% at agitation rates of 2500 rpm and 3000 rpm, respectively, compared with capsules obtained at 2000 rpm.

The elastic modulus was found to decrease by 22%, 27%, 39%, and 30% for composites with 0.5%, 1.0%, 1.5%, and 2.0% wt.% of microcapsules, respectively. On the other hand, the tensile strength increased by 33%, 20%, 8%, and 21%, respectively, at the corresponding weight percentages of microcapsules.

In terms of fatigue life, composites with a higher wt.% of microcapsules showed an improvement compared with neat epoxy. An increase of 373%, 453%, 266%, and 254% in the average number of cycles to failure (Nf) was observed at 0.5%, 1.0%, 1.5%, and 2.0% wt.% of microcapsules, respectively, at a stress level of 25 MPa. However, the Nf decreased for composites with 1.5% wt.% and 2.0% wt.% of microcapsules compared with those with 1.0% wt.% of microcapsules.

The modeling of fatigue strength coefficient (FSC) and fatigue strength exponent (FSE) by Basquin’s equation aligned with the expected behavior from the experimental results.

The self-healing capabilities demonstrated in UF-DCPD–epoxy composites make them a useful material for various types of structural applications. The healing ability of these composites ultimately contributes to the extension of the material’s lifespan. Future developments may focus on effects of temperature on mechanical characterization of the final material.

## Figures and Tables

**Table 1:** Average size of DCPD microcapsules at 2000, 2500, and 3000 rpm.

Sl. No	RPM	No. of Particles (Approx.)	Avg. Diameter (nm) (Min, Max)	Std. Deviation
1	2000-1	152	174.52 (89.2, 251.69)	43.16
2	2000-2	143	170.75 (89.03, 242.44)	34.9
3	2500	101	137.47 (73.42, 218.39)	32
4	3000	180	130.06 (73.43, 248.74)	29.28

**Table 2:** Average no. of cycles to failure (N_f_) for neat epoxy DCPD composites.

Wt.% →	0% Microcapsules		0.5% Microcapsules			1.0% Microcapsules			1.5% Microcapsules			2.0% Microcapsules		
Stress↓ (MPa)	Max Mean, Min (N_f_)	Conf. 95%	Max Mean, Min (N_f_)	Conf. 95%	% Increase	Max Mean, Min (N_f_)	Conf. 95%	% Increase	Max Mean, Min (N_f_)	Conf. 95%	% Increase	Max Mean, Min (N_f_)	Conf. 95%	% Increase
20	48,839 42,797 38,720	4244	72,457 65,032 58,934	7767	52	134,087 105,167 89,301	19,426	146	100,024 81,515 68,379	18,662	90	89,876 81,355 76,854	8355	90
25	5102 4229 3813	467	24,846 19,981 17,401	2534	373	30,258 23,401 16,025	5792	453	18,417 15,482 12,532	3244	266	16,188 14,981 13,988	1045	254
30	3635 3195 2806	341	5108 4611 3632	959	44	6486 5206 4488	863	63	4433 3777 3054	539	18	4137 3673 2811	516	15
35	1802 1568 1286	261	3092 2744 2303	340	75	2556 2382 2265	174	52	2504 2295 1959	248	46	2334 2084 1902	174	33

**Table 3:** Fatigue strength coefficient and fatigue strength exponent for composites.

Wt.% of Microcapsules	Fatigue Strength Coefficient (A)	Fatigue Strength Exponent (b)
0% (Neat epoxy)	105.94	−0.159
0.5%	128.18	−0.167
1.0%	106.45	−0.149
1.5%	103.28	−0.146
2.0%	104.96	−0.143

## Data Availability

The data presented in this study are available on request from the corresponding author. The data are not publicly available due to pending publications.
